# Reactive oxygen species in tendon injury and repair

**DOI:** 10.1016/j.redox.2025.103568

**Published:** 2025-02-25

**Authors:** Damir Kračun, Agnes Görlach, Jess G. Snedeker, Johanna Buschmann

**Affiliations:** a Division of Plastic Surgery and Hand Surgery, University Hospital Zurich, Sternwartstrasse 14, 8091, Zurich, Switzerland; b University Clinic Balgrist, Orthopaedic Biomechanics, Forchstrasse 340, 8008, Zurich, Switzerland; c Institute for Biomechanics, ETH Zurich, Gloriastrasse 37/39, 8092, Zurich, Switzerland; d Experimental and Molecular Paediatric Cardiology, German Heart Centre Munich, TUM University Hospital, Technical University of Munich, Munich, 80636, Germany; e DZHK (German Centre for Cardiovascular Research), partner site Munich Heart Alliance, Munich, Germany

## Abstract

Reactive oxygen species (ROS) are chemical moieties that in physiological concentrations serve as fast-acting signaling molecules important for cellular homeostasis. However, their excess either due to overproduction or inability of the antioxidant system to inactivate them results in oxidative stress, contributing to cellular dysfunction and tissue damage.

In tendons, which are hypovascular, hypocellular, and composed predominantly of extracellular matrix (ECM), particularly collagen I, ROS likely play a dual role: regulating cellular processes such as inflammation, proliferation, and ECM remodeling under physiological conditions, while contributing to tendinopathy and impaired healing when dysregulated.

This review explores the sources of ROS in tendons, including NADPH oxidases and mitochondria, and their role in key processes such as tissue adaptation to mechanical load and injury repair, also in systemic conditions such as diabetes. In addition, we integrate the emerging perspective that calcium signaling—mediated by mechanically activated ion channels—plays a central role in tendon mechanotransduction under daily mechanical loads. We propose that mechanical overuse (overload) may lead to hyperactivation of calcium channels, resulting in chronically elevated intracellular calcium levels that amplify ROS production and oxidative stress. Although direct evidence linking calcium channel hyperactivity, intracellular calcium dysregulation, and ROS generation under overload conditions is currently circumstantial, this review aims to highlight these connections and identify them as critical avenues for future research.

By framing ROS within the context of both adaptive and maladaptive responses to mechanical load, this review provides a comprehensive synthesis of redox biology in tendon injury and repair, paving the way for future work, including development of therapeutic strategies targeting ROS and calcium signaling to enhance tendon recovery and resilience.

## Graphical abstract

**Figure:**
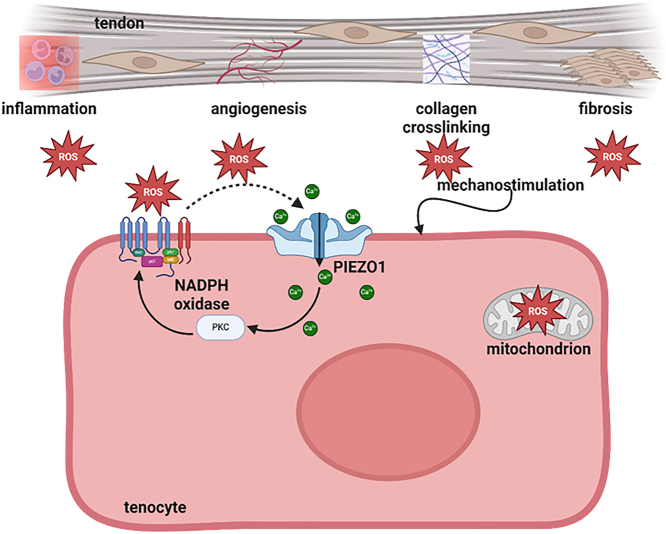

## 1 Introduction

Tendons are hypocellular, poorly vascularized collagenous connective tissues, with a primary function to enable transduction of mechanical forces from the muscle to bone [1]. Histologically, tendons mainly consist of densely packed regular connective tissue composed of sparsely distributed specialized fibroblast cells called tenocytes, which reside within a complex extracellular matrix (ECM) [2]. This healthy ECM typically forms an electron-dense, load-bearing network of uniformly aligned collagen fibrils (predominantly type I collagen) embedded within a mostly non-collagenous matrix [3,4]. The main component of the tendon ECM is type I collagen, which accounts for approximately 60–85 % of its dry mass, with the remainder made up of proteoglycans, glycosaminoglycans, glycoproteins and other collagen types (III, XII, V) [2,5]. Healthy tendons owe their immense tensile strength to the high proportion of type I collagen which in a healthy tissue accounts for 90 % of collagen and is arranged in a regular hierarchical order [6]. Following injury, type I collagen is replaced by the thinner type III collagen that rapidly forms crosslinks in an attempt to stabilize the injury [7,8]. Another striking histological property of tendons is their poor vascularity which favours anaerobic metabolism thus hampering their healing potential upon injury [9,10].

**Fig. 1:**
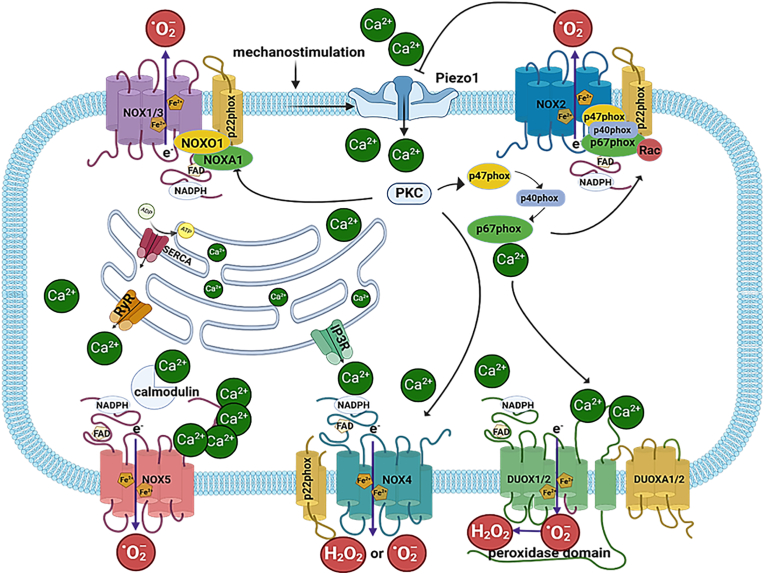
**NADPH oxidases as ROS sources and their activation by calcium.** NADPH oxidases are a family of 7 different isoforms (NOX1-5 and DUOX1&2) of enzymes producing either superoxide anion radical (O_2_^•−^) or hydrogen peroxide (H_2_O_2_). NOX1-4 isoforms require an additional transmembrane stabilization subunit p22phox, while different NOXs have different requirements when it comes to cytosolic activation factors. NOX2, requires cytosolic subunits p47phox, p67phox, p40phox and the Rac GTPase, which need to be phosphorylated by calcium activated protein kinase C (PKC) in order to translocate to the plasma membrane and join the NOX2/p22phox complex. In contrast, NOX4, although p22phox-dependent, does not seem to require any cofactors for activation; however, a role of calcium was suggested for NOX4 activation through PKC. NOX5 requires Ca^2+^ either biding it directly through its EF hands or by binding calcium-activated calmodulin to the C-terminal domain, leading to a conformational change and increased N-terminal enzymatic activity. DUOX1&2 need besides Ca^2+^ maturation factors DUOXA1&2. Upon mechanical stress cation channels (Piezo1) open facilitating the influx of Ca^2+^ that plays multiple roles in the activation of NADPH oxidases. In response to different stimuli Ca^2+^ its levels can be regulated from intracellular depots. Ca^2+^ is deposited to endoplasmic reticulum (ER) through sarco/endoplasmic reticulum Ca^2+^(SERCA), while it mainly is released from the ER through inositol 1,4,5-trisphosphate receptors (IP_3_R) and ryanodine receptors (RyR) calcium channels.

Due to their physiological role of connecting the bone to muscle and thereby transmitting force, tendons are exposed to mechanical loading in a prolonged period both in physiological, and even more so under stress (e.g. exercise) or pathological conditions. This may result in different types of tendon injury: mechanical such as tears/ruptures or inflammation as in different forms of tendinopathies. Tendon homeostatic loading facilitates a low level of ECM turnover with effective repair of microdamage, while excessive loading surpasses the intrinsic adaptive capacity of the tendon core and triggers a broader tissue response to damage with persistent mechanosensory activation until resolution is achieved. In addition, systemic conditions such as diabetes and rheumatism are known contributing factors for the development of different types of tendonitis [11,12]. Following the development of tendon injury, tendon repair is initiated and it consists of three overlapping phases: inflammation, proliferation, and remodeling (Fig. 1) [13]. In the initial, inflammatory phase, that lasts approximately 3–7 days, infiltration of immune cells, platelets and erythrocytes occurs, in which the necrotic tissue debris is phagocytosed, and the inflammation is subsequently activated. In the next, proliferative stage, lasting for 1–3 weeks, proliferation of tenocytes and their synthesis and deposition of type III collagen occurs. The last and the longest stage of the tendon repair is remodeling, which may last from several months to over as year, and is characterized by the replacement of type III collagen with more durable type I, accompanied by maturation of collagen fibers during which ECM fibers get precise and densely packed and aligned in parallel to the direction of mechanical stress [14].

Tendons are continuously exposed to oxidative stress, stemming from increased production of reactive oxygen species (ROS) and/or their reduced inactivation due to the impairment of the cellular antioxidant system [15,16]. As such, oxidative stress arising from mechanical overuse [17], ischemia/reperfusion injury [18], medicament (ab)use [19] and concurrent inflammation [20] plays a sizeable role in tendon pathology, being implicated in tendinopathies, tendon fibrosis, and tendon healing deficits (Fig. 1) [21].

Increased ROS production in tendons leads in turn to a damage of cellular compartments and macromolecules, modulation of proliferative responses, and the activation of stress responses such as unfolded protein response (UPR), DNA damage responses, apoptosis, inflammation and fibrosis all of which hamper tendon healing and recovery [22].

In this review, we summarize the current knowledge on oxidative stress affecting tendon homeostasis as opposed to tendinopathy and tendon healing. Physiology of mechanostimulation, hypoxia, inflammation and fibrosis as key processes in tendon injury and healing are covered comprehensively with regard to their redox aspects in this review. In addition, we give in detail an overview of ROS and calcium interplay in the onset of tendon injury.

### 1.1 Sources of reactive oxygen species in tendons: NADPH oxidases

ROS in physiological concentrations act in signaling capacity to maintain cellular homeostasis [23]. Oppositely, their excessive generation is deleterious due to damages to cellular macromolecular components, such as nucleic acids, proteins and lipids, thus activating different adaptive cellular stress responses that may finally lead to cell death [24]. Even though a primary ROS species, superoxide anion radical (O_2_^−•^), can be generated by any leaky oxidoreductase, usually the focus in mammalian cells is on the two sizeable sources: NADPH oxidases and mitochondria. Notwithstanding, increased ROS levels may arise from the impairment of the cellular antioxidative system and/or decreased bioavailability of natural ROS scavengers (such as nitric oxide radical, ^•^NO).

NADPH oxidases are evolutionary unique enzymes due to their sole function of producing ROS unlike other enzymatic systems that generate ROS as by-products of their main enzymatic function [25]. These enzymes are oxidoreductases which catalyze the transfer of an electron from its NADPH donor to molecular oxygen, thereby generating O_2_^−•^. These multicomponent enzymes that derive their name depending on their catalytic subunit, count seven different isoforms (NOX1-5 and DUOX1&2) (Fig. 1) [25]. Although it is clear that NOX1-3 and NOX5 are generating O_2_^−•^ as their primary product there is still an ongoing debate whether the same is true for NOX4 or does it rather produce hydrogen peroxide (H_2_O_2_) in a constitutively active fashion. One of the main arguments against this direct hydrogen peroxide production is the lack of an inner dismutation domain in NOX4, as the one present in DUOX isoforms which have been shown to produce H_2_O_2_ within the oxidase itself [25]. One of the possible explanations might be that NOX4's E-loop internally dismutates O_2_^−•^ to H_2_O_2_ [26].

NOX1-4 isoforms bind obligatory an additional transmembrane stabilization subunit, p22phox (forming the cytochrome *b*558 complex), while different NOXs have diverse requirements when it comes to cytosolic activation factors [25]. In that respect, p67phox, p47phox together with p40phox and GTPase Rac are needed for NOX2 activation [27]. NOX1 and NOX3 can function in reduced capacity with the latter [28,29]; however, they preferentially bind analogous cytosolic factors of p47phox (NOXO1) and p67phox (NOXA1) to exert their full enzymatic competency [25,30,31]. Although there are factors that amplify NOX4 function such as polymerase delta-interacting protein 2 (Poldip2) [32], protein disulphide isomerase and tyrosine kinase substrate 4/5 [33], they seem not to be obligatory for NOX4 activity [25]. Importantly, NOX4 can be regulated by calcium through protein kinase C (PKC) [34]. On the other hand, Ca^2+^-dependent EF-hand-containing NOXs such as NOX5 and DUOX1&2 need besides calcium DUOXA1&2 maturation factors as in the case of DUOX1&2 [35], while NOX5 might need calmodulin in addition to Ca^2+^ [36]. The catalytic cycle of NADPH oxidases includes five redox centers through which electrons are transferred, with NADPH being the initial electron donor and molecular oxygen being the terminal acceptor. The remaining three redox centers are contained within the NOX protein itself, and include: FAD, inner and outer heme. In the first catalytic step, electrons are transferred from NADPH to FAD, a process that is regulated by the activation domain of p67phox. In the second step, a single electron is transferred from the reduced flavin FADH_2_ to the iron center of the inner heme [25]. Since the iron of the heme can only accept one electron, the inner heme must donate its electron to the outer heme before the second electron can be accepted from the now partially reduced flavin, FADH. The potential for the transfer of the second electron, while smaller (31 vs. 79 mV), is still energetically favorable. However, the transfer of the electron from the inner heme to the outer heme is actually against the electromotive force between these two groups. To create an energetically favorable state, oxygen must be bound to the outer heme to accept the electron [25].

Reports on NADPH oxidase expression in tenocytes and tendon tissues are scarce, and one of the aims of this review is to raise awareness of this highly warranted topic at the intersection of redox and tendon biology. One of the examples is the increased expression of NOX1 and NOX4 in rat cultured tenocytes and Achilles’ tendons with collagenase-induced tendinopathy concomitant with elevated ROS levels following the stimulation with nicotinamide mononucleotide [37]. Similarly, a potent stimulator of NOX expression in tendons and tenocytes is glucose treatment [[38], [39], [40], [41]].

### 1.2 Sources of reactive oxygen species in tendons: mitochondria

In addition to NADPH oxidases, ROS are generated as by-products in mitochondrial respiration due to imperfection of the mitochondrial electron transport chain (ETC) [42]. The role of mitochondrial ROS as secondary messengers is still debated, since a signaling function has been rather attributed to ROS derived from NADPH oxidases [43,44]. Mitochondrial ETC consists of an enzymatic series of electron donors and acceptors with incremental electron affinity, in which energy resulting from electron transfer is used for pumping H^+^ through the inner mitochondrial membrane, consequently generating an H^+^ gradient which enables ATP synthase to convert mechanical work into ATP.

Electron removal during oxidative processes reduces NAD^+^ to NADH which gets oxidized by the mitochondrial complex I. In the TCA cycle, succinate is oxidized by the complex II enzyme - succinate dehydrogenase (SDH) giving fumarate and reducing FAD to FADH_2_ [45]. From complex I and II, and other sources such as electron transferring flavoprotein:Q oxidoreductase (ETFQO), proline dehydrogenase, dihydroorotate dehydrogenase, sulfide:quinone oxidoreductase (SQR), and sn-glycerol-3-phosphate dehydrogenase (G3PDH) [46], electrons are further passed to ubiquinone (Q) that gets reduced to ubiquinol (QH_2_) which is subsequently oxidized by complex III (cytochrome *bc*1 complex). Finally, the electrons are passed to complex IV (cytochrome *c* oxidase) and used for the reduction of molecular oxygen to water. This creates a transmembrane electrochemical gradient of protons (Δ*μ*_m_) which is composed of both an electrical (Δ*Ψ*_m_) and chemical (ΔpH) potential that is used by F_1_F_0_ ATP synthase for the conversion of ADP and P_i_ into ATP, a process referred to as oxidative phosphorylation [47].

**Fig. 2:**
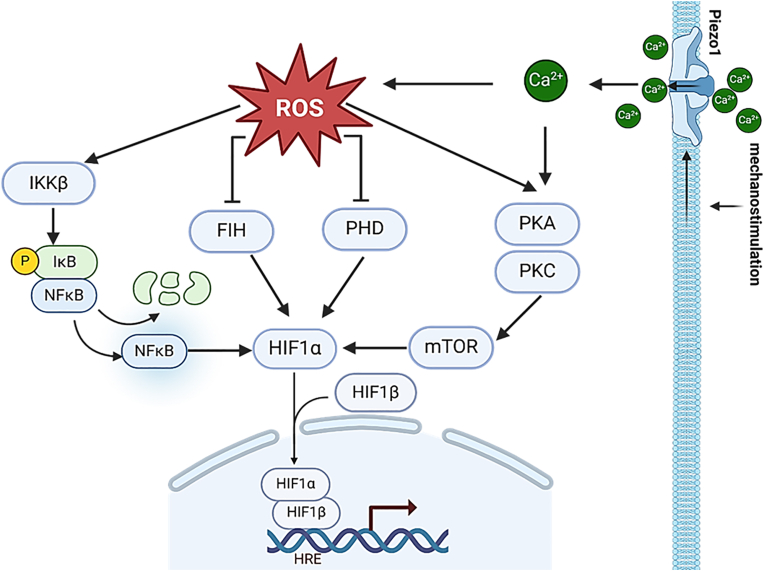
**Stabilization of hypoxia inducible factor 1 (HIF1) following mechanostimulation.** HIF1 is a α/β-heterodimeric transcription factor that consists of constitutively expressed HIF1β and a highly regulated α-subunit that initiate transcription of genes containing hypoxia response elements (HRE) in their promoters. Increased generation of ROS induces IKKβ to release NFκB from its repressor IκBα, leaving it free for an NFκB-driven HIF1α transcription. Following mechanostimulation, Ca^2+^ influx through cation channels like Piezo1 initiate the increase of ROS that on the other hand block factor inhibiting HIF (FIH) as well as prolyl hydroxylases (PHDs) contributing to HIF1α stabilization. Alternatively, Ca^2+^ signalling activates protein kinases A (PKA) and PKC stabilize HIF1α through mammalian target of rapamycin (mTOR).

Mitochondria have up to 11 different sources of O_2_^−•^ which can be divided into two different subgroups based on the source of redox potential for producing O_2_^−•^: (1) the NADH/NAD isopotential group and (2) the QH_2_/Q isopotential group [48]. The NADH/NAD isopotential group is reliant on the concentration of NADH and consists of complex I, pyruvate dehydrogenase (PDH), and branched-chain α-ketoacid dehydrogenase complex (BCKDH). The QH_2_/Q isopotential group requires reduction of Q to QH_2_ and relies on complex I, II, and III, but also the Q pool can be fed with electrons by SDH, ETFQOR, proline dehydrogenase, dihydroorotate dehydrogenase, SQR, or G3PDH [49]. Complex I and III are chief sites for mitochondrial O_2_^−•^ production (Fig. 2) [50]. Complex I inhibition by rotenone can increase ROS generation in submitochondrial particles by preventing the access of physiological ubiquinone to its reduction site thus allowing the release of one electron to molecular oxygen [51]. At complex I, O_2_^−•^ production can occur even when mitochondria are not producing ATP, due to a high proton motive force and reduced coenzyme Q pool, or when there is a high NADH/NAD + ratio in the mitochondrial matrix. Complex III funnels electrons from the CoQ pool to cytochrome *c*, and its blocking by actinomycin produces large amounts of ROS [52]. Interestingly, only sites III Qo (complex III) and G3PDH can release superoxide into the intermembrane space suggesting that these sites are of high importance for mitochondrial ROS release into the cytosol [53].

Impaired mitochondrial metabolism as observed by increased ROS, decreased superoxide dismutase (SOD) activity, cristae disorganization, and decreased number of mitochondria have been identified as mechanisms contributing to tendinopathy [54].

### 1.3 Antioxidant system and ROS inactivation

Tendons are protected against the toxicity of ROS and its metabolites by the various components of the cellular antioxidant system. This cellular defense system includes different antioxidant enzymes, redox proteins and non-enzymatic ROS scavengers. The glutathione redox system (GSH-peroxidase and GSH-reductase) inactivates ^•^NO, hydrogen peroxide, and other hydroperoxides [55]. In addition, cells harbor specialized proteins such as peroxiredoxins (Prxs), thioredoxins (Trxs), glutaredoxins (Grxs), heme oxygenases and reductases, which are also involved in cellular adaptation and protection against an oxidative assault [56]. Of antioxidant enzymes notable is the family of SODs which catalyzes the dismutation of O_2_^−•^ to molecular oxygen and hydrogen peroxide, and consists of three isoforms: cytosolic CuZnSOD (SOD1), mitochondrial MnSOD (SOD2), and extracellular SOD (SOD3, ecSOD) [57]. Another antioxidant enzyme abundantly present in the cell is catalase that converts hydrogen peroxide into oxygen and water [58]. Interestingly, an age-related increase in ROS-derived protein carbonyls concomitant with increased activity and content of Cu, Zn-SOD, and Mn-SOD, glutathione peroxidase and catalase was attenuated in tendons by dietary restrictions [58]. Not surprisingly, SOD activity and SOD levels are decreased in both rat cultured tenocytes and Achilles’ tendons with collagenase-induced tendinopathy [37]. Furthermore, SOD1^*−/−*^ mice compared to wild type littermates present a phenotype characterized by decreased collagen content and fibrocartilage mineralization associated with impaired elasticity in the supraspinatus tendon enthesis, recapitulating human rotator cuff degeneration [59]. Preservation of explanted tendons and tenocytes require mechanical loading for their culturing; hence, stress-deprivation acts unfavorably which also presents in the increase of ROS and concomitant MnSOD upregulation which might be connected to the regulation of ECM remodeling by ROS [60].

Of the peroxiredoxin family, Prdx5 expression is significantly increased in both fibroblasts and endothelial cells of degenerating human tendons [16]. In support, overexpression of Prdx5 in human tenocytes in response to hydrogen peroxide in vitro reduces apoptosis rate and enhances collagen synthesis [61]. Another enzyme family with peroxidase activity and one of the main cellular protectors against ROS excess are glutathione peroxidases whose isoform's (Gpx3) upregulation/activation induced by dexamethasone prevents both fluoroquinolone-induced and age-related tendinopathy [62].

^•^NO produced by one of three isoforms of NOS described in mammals (neuronal NOS (nNOS, NOS1), inducible NOS (iNOS, NOS2), and endothelial NOS (eNOS, NOS3)) is important in the early phase of tendon healing [63], which might relate to ^•^NO ‘s primary role of O_2_^−•^ scavenging [64]. Uncoupled NOS, an ROS source in its own right that yields O_2_^−•^ instead of ^•^NO [64], has been observed in a model of Achilles’ tendon injury and correlated with increased levels of NOS [65]. In line, the application of the NOS inhibitor N-omega-nitro-l-arginine methyl ester (l-NAME) significantly reduced cross-sectional area and failure load of the healing Achilles' tendons possibly due to increased ROS production [65]. In this context, shift in ^•^NO/O_2_^−•^ balance might be responsible for the maintenance of an optimal vascular tone in sparse tendon vessels thus contributing to the angiogenic component of the tendon repair.

### 1.4 The HIF system

Central to the cellular response to hypoxia are transcriptional factors of the family of hypoxia inducible factors (HIF) [66]. These transcription factors are α/β-heterodimers that consist of a constitutively expressed oxygen-insensitive β-subunit (alternatively termed arylhydrocarbon receptor nuclear translocator [ARNT1&3]) and a highly regulated oxygen-sensitive α-subunit (HIF1-3α) [67]. The best studied role in the cellular hypoxic response is ascribed to HIF1 that governs not only the cellular response to hypoxia but also to oxidative stress through a variety of different stimuli [68]. HIF1 (as do HIF2 and 3) binds to hypoxia response elements (HRE) in the promoter of target genes thus adjusting the cellular response to hypoxia [69]. Under normoxia, HIF1α is hydroxylated at proline residues by HIF prolyl hydroxylases (PHDs) which enables their association with the von-Hippel-Lindau protein (pVHL), an E3 ubiquitin ligase complex that sequesters HIFα for proteasomal degradation [68]. Under hypoxic conditions, PHDs are not functional due to the lack of oxygen, thus leaving stabilized HIF1α protein to associate with HIF1β assembling thus the functional transcription factor HIF1 (Fig. 2).

Tendon “normoxia” falls in the range of 1–5 % of oxygen mainly thanking to the avascular nature of this tissue [70,71]. When overloaded and/or inflamed, the oxygen demand of the tendon increases, which, combined with the tendons’ lack of blood supply, creates an oxygen-deficient environment [72]. Hypoxia and HIF1 have been described as critical regulators of early tendinopathy as increased levels of HIF1 were detected in different tendinopathy models [[72], [73], [74], [75]]. Interestingly, only recently, increased HIF1α protein stabilization was reported by Snedeker (unpublished observation) in mechanically stretched human tenocytes and murine tendons. Besides being a redox activated transcription factor, HIF1 transcriptionally regulates NADPH oxidases since HRE sequences were identified in human promoters of NOX2 [76], NOX4 [77] and Rac1 [78]. In this respect, HIF1 can act as a proxy in the ROS induction from NADPH oxidases and might form a feed-forward loop with them.

Taken together, these notions strongly support the implication of ROS from different sources stemming from mechanical and hypoxic stimuli in the development of tendon degeneration.

## 2 Tendon injury

### 2.1 Molecular mechanisms of mechanical loading injury: calcium-ROS interplay

**Fig. 3:**
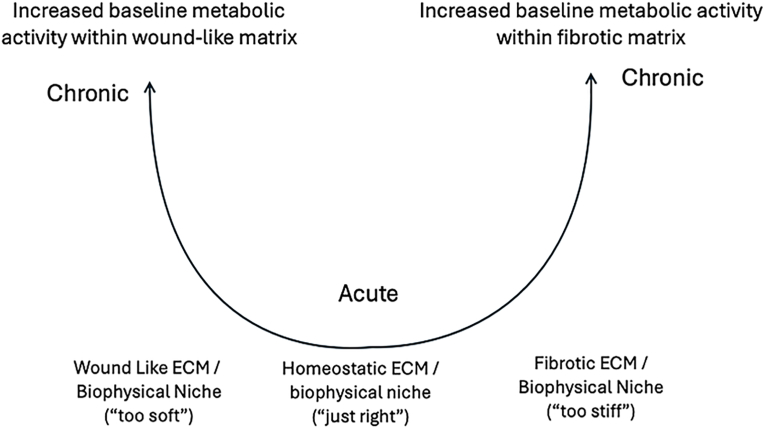
**Proposed relationship between mechanical stretching and tendon health.** The relationship between mechanical load and tendon health can be represented by a U-shaped curve, with mechanical loading on the x-axis and extracellular matrix (ECM) remodeling on the y-axis. In this case, both too little and too much load, leads to a loss of ECM homeostasis. Moderate loading promotes tendon maintenance of integrity, while overload at the cell-level (right side of the U) leads to an anabolic ECM response that can resemble fibrosis, while damage (also caused by organ overloading, but witnessed by cells as unloading of the damaged matrix) leads to insufficient cell-level loading (left side of the U) which in turn triggers matrix turnover and cell proliferation (wound healing like response).

Tendons are continuously exposed to mechanical stretching as a consequence of body locomotion. In general, the relationship between mechanical load and tendon health can be consider as represented by an U-shaped curve, with mechanical loading on the x-axis and ECM remodeling on the y-axis (Fig. 3). Here both too little and too much load is leads to a loss of ECM homeostasis. The U-shaped relationship reflects that moderate loading promotes tendon maintenance of integrity, while overload at the cell-level (right side of the U) leads to an anabolic ECM response that can resemble fibrosis, while damage (also caused by organ overloading, but witnessed by cells as unloading of the damaged matrix) leads to insufficient cell-level loading (left side of the U) which in turn triggers matrix turnover and cell proliferation (wound healing like response).

On a molecular level, mechanostimulation of the tendon enhances ROS production incremental to the degree of the observed stretching. In tensile tests of fascicles of rat-tail tendons, ROS collagen radicals were detected by electron paramagnetic resonance both by extending the duration of force application with constant force and under cyclic stretching [17]. These collagen radicals were able to produce hydrogen peroxide in reaction with water thus contributing to ROS signaling.

As a general rule, mechanical loading within the physiological parameters results in homeostatic ROS level build-up that acts beneficial by supporting the maintenance of tendon strength and heath. However, excessive tendon mechanical load is linked to exacerbated ROS generation that acts deleterious to tendon tissue homeostasis, hampering tendon health and healing.

Importantly, mechanostimulation both in its physiological or overstretching mode involves modulation of calcium levels within the tendon.

### 2.2 Calcium channels and depots

To ensure its dynamic equilibrium and a steady access of calcium as one of the basic cellular signaling moieties, cells have developed strategies for the regulation of Ca^2+^ influx/efflux through specific ion channels on the plasma membrane, as well as its release/deposition from/to intracellular depots.

On plasma membrane different classes of calcium-specific- as well as unselective channels and pumps are responsible for calcium equilibrium between the cell cytosol and the exterior. In that respect, ROS are suggested to be potent stimulators of gating of both L- [[79], [80], [81]] and T-type voltage-gated channels at least in vascular smooth muscle cells (VSMCs) [25,82]. Regarding the transient receptor potential ion channels (TRPA) family of non-selective channels, NOX2 not only colocalizes with TRPA1 but its activation also induces TRPA1 sparkles within milliseconds [25,83]. It has been reported that TRPC3 interacts specifically with NOX2 [84] to regulate calcium influx and ROS production whereas TRPC6 may actually counteract the TRPC3-NOX2 interaction, leading to different outcomes in cardiac remodeling [85]. For TRPC3 and TRPC6 family members, NOX1 was shown to be required for calcium influx [25,86].

**Fig. 4:**
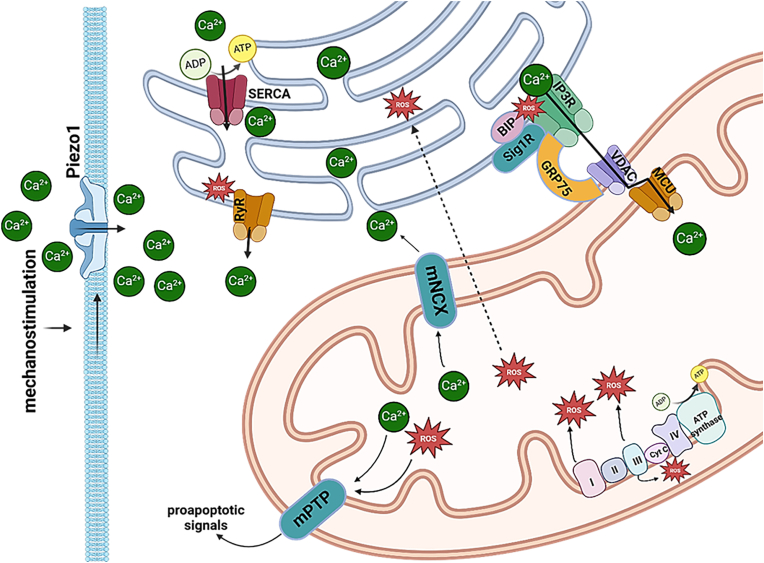
**Calcium and ROS crosstalk between endoplasmic reticulum and mitochondria following mechanostimulation.** Through a series of redox centers in the electron transport chain (ETC), mitochondria are generating a transmembrane electrochemical gradient of protons that is used by ATP synthase for the conversion of ADP and P_i_ into ATP, a process referred to as oxidative phosphorylation. Due to imperfection of this process mitochondria generate ROS at multiple sites. High levels of calcium as seen following mechanostimulation (e.g. influx through cation channels Piezo1), stimulate the ETC activity leading to higher amounts of ROS that can further target ER-based calcium channels leading to increased release of calcium and further increased ROS levels. Calcium is deposited to endoplasmic reticulum (ER) through sarco/endoplasmic reticulum Ca^2+^(SERCA), while it mainly is released from the ER through inositol 1,4,5-trisphosphate receptors (IP_3_R) and ryanodine receptors (RyR) calcium channels. These channels associate with the mitochondrial outer membrane through mitochondrial associated membranes (MAMs) complexes. These complexes consist also of the ER chaperones (BIP, Sig1R and GRP75) that modulate Ca^2+^ signaling through IP3R. Ca^2+^ from the cytoplasm enters the mitochondria through voltage-dependent anion channels (VDAC) and mitochondrial calcium uniporters (MCU) and mitochondrial sodium/calcium exchanger (mNCX). Increased ROS and calcium load can open the mitochondrial permeability transition pore (mPTP) resulting in the release of pro-apoptotic factors.

Calcium is deposited to endoplasmic reticulum (ER) through sarco/endoplasmic reticulum Ca^2+^(SERCA), while it mainly is released from the ER through inositol 1,4,5-trisphosphate receptors (IP_3_R) and ryanodine receptors (RyR) calcium channels (Fig. 4) [53]. Plasma membrane Ca^2+^ ATPase is transiently inhibited by hydrogen peroxide, while ROS stimulate the inositol 1,4,5- triphosphate (IP3) receptor (IP3R)-mediated Ca^2+^ release [87] through cysteine residue modification of the IP3R sensitizing it to IP3 [88]. Regarding the intracellular calcium depots such as SERCA, IP3 generation or application of thapsigargin, a non-competitive inhibitor of SERCA, did not alter calcium influx in VSMCs of NOX1^−/−^ mice [89]. On the other hand, RyR cannels possess oxidizable cysteines that can be targets of NOX4-or NOX1-derived ROS [90].

A non-selective mechanosensitive calcium channel that gained a lot of recognition in the last decade for its role in calcium signaling in tendons are Piezo channels [91]. Tenocytes detect mechanical forces through the activation of the mechanosensitive cation channel Piezo1 that senses shear stress induced by collagen-fiber sliding [92]. Cells depleted of Piezo1 showed a significant reduction in the shear-stress response. In support, Piezo1 knock-down in rat tenocytes isolated from tail tendon fascicles showed similar results, suggesting the importance of Piezo1 as a shear-stress sensor in tenocytes [92].

In addition, tendon stiffness is regulated by Piezo1-mediated mechanotransduction in vitro and in vivo, most likely via denser collagen cross-linking. Upon its activation, Piezo1 provides Ca^2+^ influx which might in turn regulate PKC [93]. Studies in endothelial cells showed that Piezo1's major phosphorylation site at Ser1612 also responds to shear stress and PKA and PKC activation, and its mutation abolishes the regulation of Piezo1 activity [94].

### 2.3 Role of calcium in the regulation of NADPH oxidase activation

A complex interplay between dynamically changing calcium and increased ROS events in conditions of mechanical and shear stress might provide an insight to earliest molecular events of tendon injury.

Importantly, Ca^2+^ plays a key role in the activation of NADPH oxidases both directly, as in case of NOX5 and DUOX1&2 or indirectly though the regulation of key activating enzymes (Fig. 1). PKC, a family of closely related enzymes whose activity is mediated by Ca^2+^ and diacylglycerol (DAG), is implicated in the activation of NADPH oxidases [95]. For instance, for the activation of the prototypical NOX isoform, NOX2, cytosolic factors p47phox, p67phox, p40phox and the Rac GTPase are needed to be phosphorylated by Ca^2+^-activated PKC in order to translocate to the plasma membrane and join the flavocytochrome b558 (NOX2/p22phox) complex [25,53]. In this series of events, PKC-mediated phosphorylation of p47phox seems to be an essential process for the activation of NOX2 [96]. Furthermore, the phosphorylation of p47phox reinforces the binding of its latent cytosolic partners p40phox and p67phox to flavocytochrome b558 [97]. Additionally, cooperative action of Ca^2+^ and PKC may modulate the activation of NADPH oxidases through the small Rac GPTases [98].

The assembly and activation of the NADPH oxidase complex NOX1 are initiated by NOXO1 phosphorylation at Ser154 by cAMP-stimulated protein kinase A (PKA) [99,100]. Furthermore, phorbol 12-myristate 13-acetate (PMA), a potent mitogen, has been demonstrated to activate PKC, which in turn may induce the phosphorylation of NOXO1 at Thr341/Ser154, resulting in the interaction of NOXO1 with NOXA1 and the simultaneous increase of O_2_^−•^ production [100]. Additionally, phosphorylation of NOXO1 by PKCβ is crucial for NOX1 complex activation and the concurrent increase in the rate of superoxide production [101,102]. The proline-rich region at the N-terminal end of NOXA1 can bind to the SRC downstream targets tyrosine kinase substrate with 4 SRC homology 3 (SH3) domains (TKS4) and tyrosine kinase substrate with 5 SH3 domains (TKS5), which enhances NOXA1 binding to NOX1 and causes consequent localization to invapodia and increased ROS production [25,103].

Earlier studies reported that NOX4 does not appear to require organizer or activator subunits and seemingly p22phox as a membrane located stabilizing subunit suffices NOX4 activity claiming for this reason for NOX4 to be constitutively active [104]. However, a study in skeletal muscle stem (satellite) cells showed that inactivation of the Ca^2+^ channel Piezo1 resulted in compensatory up-regulation of T-type voltage-gated Ca^2+^ channels, leading to increased Ca^2+^ influx, which strongly induced NOX4 expression via PKC [93].

NOX5 oxidase, on the other hand, contains an N-terminal regulatory domain (called NOX5-EF) with four EF-hands thus making it a direct target for Ca^2+^ activation [105]. Binding of Ca^2+^ to this domain induces conformational changes in which hydrophobic residues can now interact with the C-terminal catalytic domain and activate the enzyme [106]. In addition, NOX5 can bind Ca^2+^-activated calmodulin to the C-terminal domain, leading to a conformational change and increased N-terminal enzymatic activity [53]. Similar to NOX5, DUOX1&2 contain an EF-hand Ca^2+^-binding cytosolic region, and an N-terminal, extracellular domain with considerable sequence identity with mammalian peroxidases [107]. Important for a possible analogy with the situation in tendons, Ca^2+^ regulation of the DUOXs enzymes might be crucial for the recruitment of immune cells, foremost leukocytes, to the site of injury. For instance, Ca^2+^ flashes have been shown to trigger DUOX1-dependent hydrogen peroxide in zebrafish after mechanical injury and subsequently the recruitment of leukocytes to migrate to the wound [108].

The picture gets even more complex since Ca^2+^ channels and PKC might be reciprocally regulating one another [109]. NADPH oxidase-derived ROS may further elevate intracellular Ca^2+^ by enhancing membrane ion channel activity and/or promoting Ca^2+^ release from intracellular stores [110].

### 2.4 Role of calcium in mitochondrial ROS production

The interplay between mitochondrial metabolism responsible for ROS generation and calcium is complex, but it is generally believed that the metabolic state of the mitochondria determines the effects of Ca^2+^ on mitochondrial ROS levels. A well postulated role of Ca^2+^ in mitochondria is the promotion of ATP synthesis by stimulation of TCA enzymes thereby increasing the consumption of oxygen and resulting in increased ETC electron leakage and ROS build-up [111]. Increased Ca^2+^ levels are responsible for the diminished ROS build-up from both complexes I and III, while they can enhance ROS generation when these complexes are inhibited by pharmacological agents [53]. When there is no ongoing ATP synthesis (high membrane potential), Ca^2+^ uptake results in decreased ROS generation [112]. In the case of mitochondrial membrane depolarization, respiratory inhibition and generation of ROS, together with a massive release of matrix Ca^2+^ were observed that leads to swelling of mitochondria and the outer mitochondrial membrane breaching thus compromising mitochondrial integrity. To add insult to injury, a voltage and Ca^2+^-dependent mitochondrial permeability transition pore (mPTP) opens prompting cell's demise [113].

Shear or mechanical stress creates besides increased ROS production also a hypoxic microenvironment which adds to activation of HIF1 as was shown in a variety of cells [68,114], and might by analogy act similarly in tendons. In this respect, HIF1 might have a crucial role of integration of mechanical stress within tendons being activated both via calcium and ROS signaling. In support of this assumption are observations of specific calcium channels and pumps regulating HIF1 activity through controlling its transcription, translation, stabilization, or nuclear translocation in cancer cells [115]. More recently, Snedeker et al. suggested that Ca^2+^ activation of HIF1 in tendons acts via mTOR (unpublished observation) analogous to HIF1 activation in a mouse model of heterotopic ossification [116].

Due to HIF1's multi-facetted and versatile action, it is probably impossible to separate its role in the conductance of mechanical/shear stress in the context of tendon injury from its almost canonical role in inflammation in the same context which is discussed in the corresponding section of this review.

### 2.5 Diabetic tendon injury and repair

Numerous preclinical and clinical evidence identified type 2 diabetes mellitus (T2DM) as a significant risk for tendon injury occurrence and/or impairment of tendon healing since it causes structural, inflammatory, and vascular changes in the tendon [117].

In diabetic tendinopathy, the degeneration mechanistically arises from advanced glycation end-products (AGE) [118] that accumulate in load-bearing collagen [119,120]. AGEs are chemically heterogeneous end products of non-enzymatic protein glycation prevalently encountered in tissues with low turnover proteins, such as collagens in the ECM of articular capsules, ligaments and muscle-tendon units [121,122]. They may contribute to the supraphysiological increase in collagen cross-linking which alters the mechanical properties leading to a decrease in elasticity and tensile strength, and an increase in mechanical stiffness [121,123,124]. Upon binding to their receptor (RAGE), AGEs mediate their effects by promoting oxidative stress, inflammation, and apoptosis [125]. AGEs’ versatile signal transduction cascades and downstream pathways include also the induction of ROS generation from NADPH oxidases [122] and mitochondria [125].

In patients with diabetes suffering from rotator cuff tears (RCT) staining for AGEs and RAGE as well as NOX expression, ROS levels, collagen type I and inflammatory cytokines (i.e., IL6 and IL1β) expression and apoptosis were increased [126]. Treatment with high AGE significantly decreased viability and tensile strength of human rotator cuff-derived cells accompanied by increased ROS and HIF1-driven VEGF secretion [127]. In rat diabetic Achilles’ tendons or high glucose (HG)-treated tenocytes derived from them, hyperglycemia caused disorganized collagen fiber arrangement, augmented ROS levels concomitant to increased expression of NOX1 and NOX4 mRNA, MMP2, TIMP2, and collagen III expression additionally inducing a pro-inflammatory state as seen by increased levels of IL6 [[38], [39], [40], [41]]. In contrast, pretreatment with the unspecific NOX inhibitor apocynin [40] or the unspecific flavonoid ROS scavenger quercetin [38] or with the adrenal steroid dehydroepiandrosterone (DHEA) [39] prevented these responses. Interestingly, addition of AGE to RCT patient tenocytes increased ROS levels concomitant with the expression of NOX1 and NOX4, IL6, and RAGE, all of which were decreased by co-treatment with apocynin [128]. Interestingly, hyperglycemia and/or aging induces ROS from NOX1 that forms a positive feedback loop with IL6 in both in human and murine endothelial cells [129].

Clinical studies have also indicated that, similar to hyperglycemia, increased fat load such as in hypercholesterolemia represents an independent risk for tendinopathy [130]. Increased hydroperoxidized LDL plasma levels, possibly due to increased ROS, were reported in a study with patients having combined familial characteristics such as hypercholesterolemia and moderate grade thickness of the Achilles' tendon [131]. In support, in Achilles’ tendons from high-fat diet fed apoE mice the expression of tendon cell markers (collagen I, scleraxis, tenomodulin) was lowered, possibly due to elevated ROS levels via the inflammatory/NFκB axis [132]. In addition to the latter, cholesterol application increased ROS and expression levels of NOX4 and catalase in tendon-derived stem cells while N-acetyl-l-cysteine (NAC) pretreatment abrogated the action probably through the blockage of cholesterol-induced phosphorylation of IκBα [132]. Besides acting through the NFκB pathway, cholesterol overload triggered ROS generation through the AKT/FOXO1 axis, while the ROS scavenger NAC and the FOXO1 inhibitor AS1842856 blocked cholesterol-induced activation of this pathway rescuing the TDSCs from cholesterol-induced apoptosis and autophagy [133].

Hyperglycemia and its AGE products, as well as hyperlipidemia increased ROS consistently in different in vitro and in vivo metabolic tendinopathy models [134]. It may be that NOX1 and NOX4 [38] oxidases are crucial for tendon ROS generation following metabolic perturbances, thus rendering them important for the increased inflammation and ECM remodeling that are readily encountered in the process of tendon healing. Mechanistically ROS production and inflammation seem to be mediated mainly through NFκB and FOXO1 pathways.

These results point that the control of ROS production and breakup of persistent inflammation, could be a plausible therapeutic target for combatting the diabetic tendinopathy.

## 3 Tendon healing

Tendon injury and the subsequent tissue responses rely on cellular processes such as: cytokine secretion, modulation of programmed cell death, chemoattraction and migration, and the synthesis and breakdown of ECM components. These processes act as part of the tissue stress response that includes inflammation, angiogenesis and ECM remodeling aiming to promote tendon repair and tendon healing. In all of these processes timing and the amount of ROS are detrimental for the successful healthy healing of the tendon.

### 3.1 Proliferation and self-renewal

Tendon stem/progenitor cells (TSPCs) or tendon-derived stem cells (TDSCs) were isolated and described in human and mouse and show characteristics of stem cells such as multipotency, clonogenicity, and self-renewal capacity [135,136], and as such hold a great potential in tendon healing since they can differentiate into functional tenocytes [137]. Phenotypically, fully developed specialized cells of the tendon tissue are tenocytes known also as tendon-specific fibroblasts or fibrotenocytes and constitute up to 95 % of the tendon cellular mass [138]. The ongoing debate on the authenticity of TSPCs, although outside of the scope of this review, is important to be highlighted since the lack of accurate distinction between and TDSCs, might in some instances obscure the data interpretation [136].

TSPCs are often exposed to oxidative stress at tendon injury sites, which impairs their physiology as well as therapeutic application [139]. High local concentrations of ROS inhibit the activity of transplanted stem cells and hinder tendon repair [140]. Proliferation of fibrotenocytes, tenocytes as well as self-renewal, migration and differentiation potentials of TSPCs are crucial for tendon repair following an injury [136].

Supplementation with exogenous ROS such as H_2_O_2_ affects both recruitment and survival of TDSCs, moreover reducing colony formation and suppressing cell migration, cell viability, and proliferation [141]. Decreased ROS production following pretreatment with NAC mitigated the pro-proliferative responses in TSPCs promoting their survival and differentiation thus facilitating tendon repair after injury in rats [142]. Physiological ROS concentrations act pro-proliferatively and promote mesenchymal stem cell (MSC) proliferation and migration by activating the ERK1/2 and Jun1/2 pathways and mediated by the NOXs family [141]. The effects of antioxidant supplementation on the proliferation of tendon-specific cells seems to be dose dependent, with lower doses promoting it and higher decreasing it [143].

Another class of anti-inflammatory drugs, glucocorticoids (GC), has a multifaceted action on ROS production especially in non-inflamed healthy tissues and cells, with lower doses inducing ROS from NOXs and mitochondrial sources as well as HIF1α levels and HIF activity in endothelial cells [144]. Similarly, high doses of the synthetic GC dexamethasone exerted deleterious effects on tenocyte proliferation while co-treatment with vitamin D relaxed this phenotype by decreasing ROS levels and acting pro-proliferatively by phosphorylation of extracellular signal-regulated kinase and p38 MAP kinase [145]. Possibly, the role of vitamin D lies in the regulation of Ca^2+^ absorption and is the key process in this instance [146]. Dexamethasone treatment induced ROS generation while reducing viable tenocyte number and proliferation, activating the stress-response transcription factors FOXO1 and FOXO3A and decreasing the phosphorylation of their negative regulators, the pro-proliferative kinases ERK and PKB/Akt [147]. In vitro, adipose stem cell-derived exosomes delivery significantly improved GC-suppressed cellular proliferation, migration, contributing to ECM build-up by increasing tenocytic matrix molecules and decreasing degradative enzyme inhibitors [148]. In vivo studies revealed that additional adipose stem cell-derived exosome injection restored impairments in histological and biomechanical properties owing to GC administration [148].

Delivery of bone marrow MSCs either to H_2_O_2_-injured tenocytes in vitro or in vivo to Achilles’ tendon in a rat collagenase I-induced tendinopathy model restored tenocyte mitochondrial function and promoted their proliferation and resistance to apoptosis concomitant to ROS decrease [149]. Interestingly, the migration and proliferation of tendon cells were reduced after HIF1 inhibition, as well. Moreover, HIF1α levels increased in tendinopathy in vivo and in tenocytes in vitro, while HIF1 inhibition alleviated the severity of tendinopathy by blocking NFκB and MAPK signaling pathways [150].

### 3.2 Activation of cell stress pathways and cell death

Mechanistically, increased ROS load is associated with loss of TSCP's pro-proliferative phenotypes due to replicative senescent processes [151]. High dose resveratrol (30 μM), a polyphenolic plant-based compound with antioxidant properties alleviated dexamethasone‐induced tendon senescence by activating the p53 deacetylase Sirtuin1 thus forestalling the p53/p21 senescence pathway [152]. In support of the senolytic properties of resveratrol, it promoted osteogenic differentiation of senescent bone mesenchymal stem cells (BMSCs) by enhancing mitochondrial function and inhibiting AMPK pathway and ROS production [153,154]. On the other hand, dexamethasone was shown to be a potent inducer of ROS and the HIF system at least in endothelial cells [144]. Opposing, resveratrol at lower concentrations (<10 μM) was shown to act as a senomorphic and/or antioxidant, preventing cellular senescence; whereas at higher doses, it mainly shows its pro-oxidant, or even induce senescence [152,155].

Oxidative stress leads also to protein damage and the activation of the unfolded protein response (UPR) that might lead to apoptotic death of tenocytes. Activation of mitochondrial aldehyde dehydrogenase 2 (ALDH2) might alleviate oxidative stress and ER stress. Treatment of Alda-1, a potent activator of ALDH2, prevented H_2_O_2_-induced oxidative stress and depolarization of mitochondrial membrane potential in tenocytes [156]. Besides decreasing mitochondrial ROS load, application of Alda-1 prevented the ER stress-mediated apoptotic cascades and upregulation of the inflammatory cytokines IL1β and TNFα in cultured tenocytes [156].

Oxidative stress occurring in the hypovascularized chronic RCT due to hypoxia and ROS accumulation resulted directly in unregulated autophagy or in autophagy mediated through HIF1, mTOR, and UPR [22,157]. Autophagy was induced in hypoxia predominantly via HIF1/Bcl2-interacting protein 3, (BNIP3) thus releasing Beclin1 [22].The hypoxia mimetic CoCl_2_ induced autophagy in mouse C2C12 myotube cells [158], with a concomitant increase in the autophagosome marker LC3II, and a decrease in p62, an autophagic adapter protein degraded during increased autophagy [159,160]. Exposure to hydrogen peroxide increased the expression of LC3II, Atg5-12 and Beclin1 as well as GFP-LC3 puncta formation, and the number of autolysosomes pointing to autophagy persistent even in the presence of the apoptotic inhibitor zVAD [161]. However, treatment with the natural antioxidant cyanidine reduced H_2_O_2_-induced cell death, LC3II expression, GFP-LC3 puncta formation, and autolysosomes, as well as Atg5-12 and Beclin1 expression [161]. The enhanced autophagy in RCT leads to tissue destruction with differences reflecting the chronicity. Indeed, in patients, chronic RCT injury induced a higher ROS production than the acute one concomitant with the higher expression of Beclin1 and downregulated expression of mTOR [162].

Infusion of platelet rich plasma (PRP) decreased the expression of ECM-related genes and induced ROS and the expression of autophagy-related genes such as Atg10, Bnip1 and GABARAPL2, without affecting Beclin1 or Trim13 expression [163]. It might be that autophagy is a rescue response to supraphysiological concentration of ROS, in this case coming from the stimulation with PRP, that synergistically with inflammation act pro-preservant on the tissue integrity.

In line with enhanced proliferation of tendon-specific cells, resistance to programmed cell death of these cell types is also detrimental for tendon repair [164]. H_2_O_2_ significantly impaired TSPC proliferation and tenogenic differentiation capabilities, due to increased intracellular ROS accumulation and promotion of apoptosis [139].

In patients with rotator cuff or biceps tendinopathy, mitochondrial membrane potential was decreased in samples of degenerative tendon, while the expression of BNIP3, implicated in pro-apoptotic pathways and mitochondrial dysfunction was upregulated [165].

### 3.3 Role of ROS in proteolytic extracellular matrix remodeling in tendons

Tendon degeneration is characterized by intensive ECM remodeling that includes: fragmentation of collagen fibers, increased expression of matrix metalloproteinases (MMPs), accumulation of proteoglycans, increased neoinnervation and neovascularization [143]. In this respect, the ECM composition reflected through the synthesis of collagen and other ECM components as well as their crosslinking and degradation might be a detrimental factor for successful tendon healing, processes in which ROS play an important role [3,4]. Along those lines, analyses of stromal tissue transcriptome and secretome revealed that an elevated tissue oxygenation and temperature of stromal niche drives a ROS-mediated cellular stress response prompting an immune-modulatory phenotype within the degrading stromal tissue [3,4]. One of the striking histological features of a healthy tendon is its densely packed ECM mainly comprised of type I collagen that accounts for 90 % of collagen and is arranged in a regular hierarchical order [6]. Healthy tendons owe their immense tensile strength to the high proportion of collagen I; however, following tendon injury this sturdy collagen type I gets replaced by the thinner collagen III that rapidly forms crosslinks in an attempt to stabilize the injury [7,8]. ROS exert direct effects on ECM integrity inducing crosslinking and increasing the content of collagen III over type I. Additionally, ROS shows its vital roles in ECM degradation gating a complex proteolytic breakdown of the functional collagen backbone [3,4]. In the initial phases of tendon damage collagen III is produced as a conserved mechanism to provide a rapid ‘patch’ to the area of damage [2]. These changes in the ECM composition loosen its consistency, enabling the migration of immune cells to the damaged area concomitant with the promotion of angiogenesis and tenocyte proliferation necessary for the early stage of healing.

Electron-paramagnetic resonance (EPR) spectroscopy of stretched rat tail tendon showed that mechanical stress on collagen produces collagen mechanoradicals which can be envisaged as a yet undiscovered source of oxidative stress, finally converting into hydrogen peroxide assuming their signaling role maintaining the redox homeostasis [17]. Interestingly, collagen mechanoradicals have a highly conserved accumulation of ROS-scavenging residues (Tyr/Phe/Met) around collagen crosslinks, which might suggest that force-induced oxidative stress might be an evolutionary adaptation for collagen I [17]. The latter study provides a compelling set of evidence that suggests an additional role for collagen I as a radical sponge against mechano-oxidative damage.

Exogenous ROS, such as H_2_O_2_ decreased collagen synthesis [61]. In line, supplementation with l-arginine [166], as well as with NAC [142] increased the expression of collagen Ia, and prevented ECM degradation. Additionally, ROS are exerting their role on ECM remodeling in the tendon by enhancing cytokine expression. Cytokines exhibit various effects on tendon ECM remodeling, with: IL6 increasing the total collagen synthesis [167], TNFα decreasing collagen I deposition [168], IL4 knockout enhancing collagen disorganization and lower crosslinking [169], while IL33 and IL17 act more discretely by increasing the ratio of type III to type I collagen and the upregulation of MMP production [2]. Although, IL33, a marker of early tendinopathy, induced dose- and time-dependent upregulation of total collagen protein, its main action was the promotion of synthesis of collagen III over collagen I thereby hastening the repair [170]. IL33-mediated changes in collagen expression were abrogated by NFκB and ERK inhibition, suggesting that IL33 operates in tenocytes via a canonical IL1 receptor (IL-R) signalling pathway [170]. In vivo, single peritendinous injections of bupivacaine caused apoptosis in endotendon cells and an increase of pro-MMP9 shifting the collagen ratio towards collagen III while almost abrogating scleraxis mRNA expression [171].

On a ECM therapy note, HeLa and Vero cells grown on dishes coated with two cryptic bioactive peptides isolated from bovine tendon collagen: C2 (with cell adhesive properties) and E1 (with cell adhesive and antioxidant properties) might have a potential in countering external ROS stress acting pro-survival and imparting cytoprotection in mammalian cell systems [172].

### 3.4 Inflammation

Inflammation plays a pivotal role in tendon healing, creating an inflammatory microenvironment through cytokines and immune cells that aid in debris clearance, tendon cell proliferation, and collagen fiber formation. However, uncontrolled inflammation can lead to tissue damage, and adhesions, and impede proper tendon healing, culminating in scar tissue formation. In this respect one has to discriminate between transient acute inflammation in response to tendon injury that ameliorates tendon healing, and chronic inflammation that is either a result of an impaired tendon healing process or a systemic condition that results in persistent tendonitis.

Timely switch on/off of inflammation mediated by cytokines is responsible for governing specifically pro- and anti-inflammatory stages of the initial phase of tendon healing [173].

The first responders to tissue injury or infection are circulating and/or tissue resident innate immune cells that cause acute local inflammation. These cells have evolutionary developed mechanisms for detecting an infection or cell damage, such as pattern recognition receptors (PRR) that recognize pathogen associated molecular patterns (PAMP) present on microbes and/or damage associated molecular patterns (DAMP) or “alarmins” – proteins released from cells upon infection, necrosis, or injury [174].

Mammalian cells have five classes of PRRs, with a classic example being the membrane-bound class of toll-like receptor (TLR) family that comprises at least 13 functional members expressed on the cell surface and intracellularly in endosomal compartments [175]. Following pathogen detection by means of ligand biding, five adaptor proteins containing a Toll–interleukin 1 (IL1) receptor (TIR) domain bind(s) to the cytosolic TIR domains of TLRs. Five TIR domain–containing adaptors are involved in propagating TLR signaling (for a review readers are referred to Ref. [176]): MyD88 (myeloid differentiation primary-response gene 88) - the universal adaptor for all TLRs except TLR3, TIRAP/MAL (MyD88-adaptor-like protein) - required for the recruitment of MyD88 to TLR2/4, TRIF [TIR-domain-containing adaptor protein inducing interferon β (IFNβ)] - used by TLR3 leading to activation of IRF3 through TBK1 (TRAF-family-member-associated NFκB activator-binding kinase 1), TRAM (TRIF-related adaptor molecule) - used only by TLR4, and its main function is recruitment of TRIF, whereas SARM (sterile α- and armadillo-motif-containing protein) inhibits TRIF-dependent signaling [176].

MyD88-dependent signaling triggers downstream phosphorylation of interleukin 1 receptor–associated kinase1/4 (IRAK1/4), that together with transforming growth factor β–activated kinase 1 (TAK1) are essential for the activation of NFκB, interferon regulatory factor 1/5/7 (IRF1/5/7), as well as TLR-independent signaling activated by IL1/18 and IFNγ [176]. Essentially, MyD88-dependent pathway is crucial for transducing inflammatory signals from interleukin receptors, stimulates downstream NFκB signaling and proinflammatory cytokine production [177]. TLR4 induces two distinct signaling pathways controlled by the TIRAP-MyD88 and TRAM-TRIF pairs of adaptor proteins, which elicit the production of proinflammatory cytokines and type I interferons, respectively [178]. This TLR4's dual activation seems to be sequential, with first inducing TIRAP-MyD88 signaling at the plasma membrane and is then endocytosed and activates TRAM-TRIF signaling from early endosomes [178].

In this way they initiate signal transduction that culminates in the canonical activation of NFκB, IRFs, or MAP kinases which regulate the expression of proinflammatory cytokines, chemokines, and type I IFNs thus ultimately protecting the host from microbial infection or injury [179]. The release of these pro-inflammatory mediators besides directly inducing inflammation can also act indirectly through promotion of inflammatory T cells [180]. A signaling component that integrates the different PRR pathways for NFκB activation is TAK1 [180], and this stringent control of NFκB and MAPK signaling is critical during innate immune responses [181]. ROS have been identified as activators of MAPK signaling, including JNK, p38, and ERK pathways [182,183]. Hydrogen peroxide-induced phosphorylation of MAPKs and NFκB through TAK1 activation in bovine synovial fibroblasts resulted in increased COX2 and PGE2 expression [184].

**Fig. 5:**
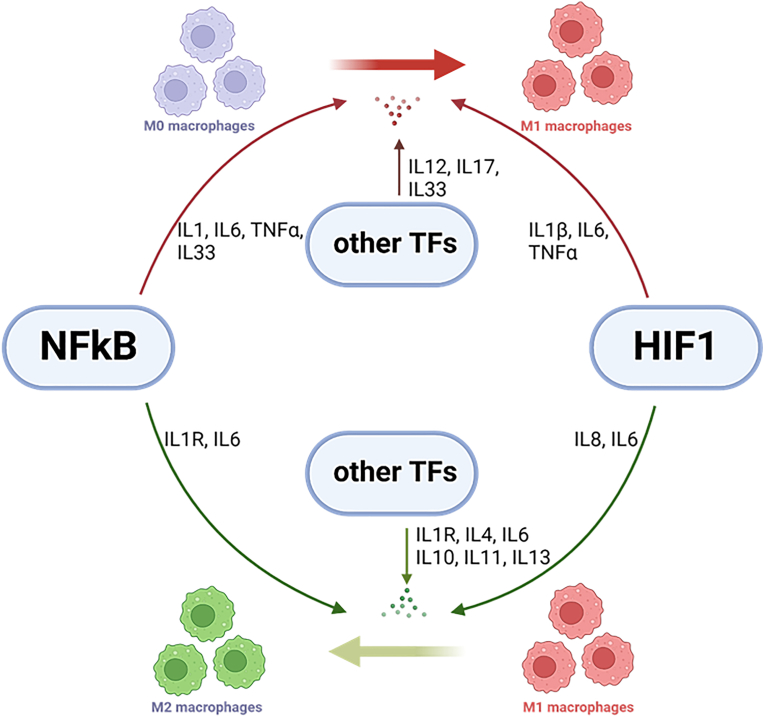
**Macrophage polarization in tendon injury.** Proinflmmattory cytokines (IL1, IL1β, IL6, IL12, IL17, IL33, TNFα) governed by NFκB, HIF1 or other transcription factors (TFs) mediates transition of naive macrophages (M0) to inflammatory macrophages (M1) promoting a proinflammatory state. Opposing, resolving of inflammation is characterized by the polarization of M1 to antiinflammatory macrophages (M2) by IL1R, IL4, IL6, IL8, IL10, IL11, and IL13 that promote tissue-repairing state.

In the process of tendon healing, inflammation in connection with increased ROS levels, is necessary for the recruitment and infiltration of immune cells (Th1 T cells, neutrophils, and M1-type macrophages) with a primary goal to eradicate necrotic cells debris and to set the stage for the recovery phase of the healing characterized by tenocyte proliferation and collagen production [173]. The inflammatory environment subserves several important functions in the early onset of inflammation such as ECM remodeling, chemoattraction, motility and proliferation of immune cells, and promotion of angiogenesis. The M1 macrophage phenotype appears to be a primary driver of the early inflammatory process in the healing tendon since their number is largely elevated in the first two weeks of healing and they localize to areas of newly formed tendon tissue as well as to the areas of tissue resorption [185]. These cells release ROS and proinflammatory cytokines and mediators such as IL1, IL6, IL12, and TNFα (Fig. 5) [186]. In the acute phase response to stress, satellite cells in muscle and dermal fibroblasts in skin are activated and recruited by IL6 [[187], [188], [189]]. Moreover, IL6 plays a vital role in activating reparative fibroblasts within the extrinsic tissue compartment (i.e. epitenon or paratenon) and recruitment of these cells to the damaged tendon core tissue in non-sheathed tendons [190,191]. While this likely represents a critical step in normal tendon healing, it is suggested that extended and excessive IL6 signaling may causally exacerbate tendinopathy in non-sheathed tendons [192,193]. Interestingly, IL6 has been shown to upregulate NOX1 in response to high glucose treatment in endothelial cells [129].

Inflammation accompanied by the increased number and metabolic activity of immune cells together with an increased oxygen demand of tenocytes and tendons’ lack of blood supply creates a local oxygen-deficient environment. On the other hand, increased oxygen metabolism results in increased ROS that together with the hypoxic inflammatory microenvironment increase the stabilization and activation of HIF1 [68]. HIF1 participates in the regulation of inflammation and is expressed in several types of immune cells in response to stress, including macrophages, dendritic cells, neutrophils, and Th17 cells [194]. HIF1 responsive genes range from oxygen transporters, glycolytic enzymes, angiogenic modulators, genes of the immune system such as the TLR receptor family [195] to diverse panels of proinflammatory cytokines. HRE sequences are present in IL6 [196], IL1β [197], COX1 and COX2 [198]. Both COX1&2 isozymes catalyze the conversion of arachidonic acid to endoperoxide intermediates, which are ultimately converted to prostanoids such as PGE2 [198,199]. Similarly, the NSAID licofelone that inhibits arachidonate 5-lipoxygenase (5-LOX), an initiator of leukotriene biosynthesis, increased the load to failure of repaired tendons and increased the force production of muscle fibers improving tendon repair through the inhibition of COX1, COX2, and 5-LOX following healing of chronic RCT in rats [200]. In support, COX2 is reported to be a target gene of HIF1 in several cell lines including osteoblasts [201] as well as to enhance the neovascularization of inflammatory tenocytes through the HIF1/VEGFA/PDGFB axis [202]. The selective COX2 inhibitor Celecoxib impaired tendon-to-bone healing, weakened mechanical stability and decreased PGE2 content of the synovial fluid following anterior cruciate ligament reconstruction [203].

Besides directly promoting collagen crosslinking, ROS can also indirectly shape ECM through enhanced expression of proinflammatory cytokines. Pro-inflammatory cytokines can exhibit various effects on tendon ECM remodeling. For example, IL6 can increase total collagen synthesis [167]; IL17 and IL33 can shift the ratio of type III to type I collagen and up-regulate MMP production [2]. Both in rat cultured tenocytes and in Achilles’ tendons with collagenase-induced tendinopathy levels of IL6, ROS and NOX1 and NOX4 were increased while SIRT1 and SIRT6 were decreased together with SOD activity [37]. Similarly, in the RCT model, elevated nerve growth factor (NGF) protein expression that is under the regulation of the inflammatory mediator TNFα, induced atrophy in the supraspinatus muscle tissue with a concomitant increase of IL1β, COX2, oxidative stress and apoptosis [204]. TNFα, IL1β, and ROS in RCT groups all rose up significantly 24 h postop demonstrating a correlation between postoperative inflammation and oxidative stress. TNFα inhibition resulted in decreased NFG expression, decreased tissue fibrosis, and improved tendon atrophy. Moreover, inhibition of TNFα reduced the expression of IL1β and COX2 and decreased apoptosis and oxidative stress [204].

Clearly, exacerbated ROS drive the inflammatory response in tendon injury that is further perpetuated by an ROS-inflammation feed forward loop. For a healing process to progress, this loop needs to be resolved by the influx of anti-inflammatory cytokines since prolonged exposure to proinflammatory cytokines can lead to tendon adhesions and hinder tendon healing. For this to happen, ROS levels have to decrease and processes such as angiogenesis and immune cells recruitment to halt. In order to prevent the excessive proinflammatory response in the phase I, anti-inflammatory signaling through the secretion of IL1 receptor antagonist, IL4, IL13, IL33 or TGFβ is activated from damaged cells and facilitates the conversion of resident macrophages to M2 phenotype [205,206]. The release of IL33 triggers downstream responses from macrophages, Tregs, and other intrinsic immune cells [207], that produce IL10, which acts as an important anti-inflammatory factor to resolve inflammation, similarly to IL4 [208]. IL4 is critical for wound healing in various tissues, recent studies have sought to elucidate potential roles for IL4 in tendon repair [173]. IL4 and IL13 have been shown to convert resident macrophages into a M2 phenotype - that later in the healing process stimulate fibroblast proliferation and new tissue deposition [209].

In summation, unravelling the specific mechanisms of inflammatory signaling in tendinopathy is of great significance for exploring the potential anti-inflammatory therapeutic avenues.

## 4 Therapy

The therapeutic approach for tendon healing and repair should include promotion of tenocyte differentiation and proliferation, ECM remodeling, new collagen synthesis and deposition, while ideally decreasing pathological ROS levels, forestalling or inhibiting inflammation, fibrosis and apoptosis. These processes are significantly regulated both in their pro-injury and pro-healing states by ROS; hence, taming their production and fine-tuning of their spatio-temporal appearance and scavenging might be a plausible main- or supplementary therapeutic option. As we argue in this review, ROS play a significant role in all of the latter processes, hence their precise temporal taming presents a viable therapeutic option that was employed in a plethora of studies. In support, accumulating evidence supports that supplementation with antioxidants or natural compounds with antioxidative properties might provide preventive and curative effects for tendinopathies of various etiologies and also ameliorate tendon tear or rupture injuries [143].

A major problem following tendon injuries, especially of laceration type is the low mechanical strength of the repaired tissue [210]. Of the specific tendon injury complications, peritendinous adhesion is one of the most common negatively impacting the healing process [211]. One viable strategy for improving the functional and biomechanical properties of ruptured tendons is the topical delivery of medicaments at the injury site [210]. Specific anatomical position of tendons allows external topical delivery of medicaments. For these purposes different alloy composition some of which some possess antioxidant properties themselves combined with a variety of delivery systems have been tested.

Several approaches have been devised to prevent peritendinous adhesion, such as surgical tenolysis, systemic and local drug administration, postoperative physical therapy, and the use of physical barriers or their combination [211]. Among these, the electrospun membranes (EM) of different composition are widely favored for an anti-adhesion strategy [210] ESP serve not only as a physical barrier and defense against pathogens, but they also inhibit myofibroblast proliferation and invasion into the repaired tendon site while allowing nutrient exchange through a microporous structure [211].

### 4.1 Therapy with antioxidants and medicaments

In both immature and young adult tenocytes, quinolones induced significant decreases in intracellular reduced glutathione levels, and ROS production, as compared to controls; however, it is not clear whether ROS production was a limiting factor or just a component of the tenotoxicity [212,213]. Elevated ROS are also linked to the tenotoxic response to fluoroquinolone in vitro [212,213]. Rat tendons systemically and solely treated with fluoroquinolone exhibited mechanical fragility, increased number of cells with round nuclei, and DNA damage all of which are commonly encountered in tendinopathy; only co-treatment with dexamethasone attenuated these effects and increased the expression of Gpx3. Similarly, anthocyanins with antioxidant properties decreased hydrogen peroxide-induced activation of ERK1/2 and JNK, and reduced the production of ROS thus dose-dependently inhibiting apoptosis in cultured tenofibroblasts [214]. The expression of antioxidants, such as Prdx5 in fibroblast and endothelial cells in tendons, provided protection from apoptosis and cell function loss [16]. Similarly, overexpression of Prdx5 overcame negative effects of H_2_O_2_ in human tenocytes by reducing the apoptosis rate [61]. Additionally, NAC rescued TDSCs from cholesterol-induced apoptosis and autophagy [133].

Treatment of cultured rat Achilles' tendon-derived cells with the local anesthetic bupivacaine exerted effects on cell viability that could be counteracted by NAC or reduction of extracellular calcium [171]. Nicotinamide mononucleotide (NMN), a precursor of nicotinamide adenine dinucleotide important for the maintenance of redox homeostasis, increased the expression of SIRT1 and SIRT6 as well as SOD activity in cultured rat tenocytes and Achilles’ tendons with collagenase-induced tendinopathy, but decreased the levels of NOX1, NOX4, IL6, ROS, and apoptosis demonstrating the antioxidative effects of NMN [37].

Pretreatment with NAC or reduction of extracellular calcium of cultured rat Achilles’ tendon-derived cells with bupivacaine increased cell viability acting anti-apoptotic [171]. In vivo, single peritendinous injection of bupivacaine caused apoptosis in endotenon cells and an increase of pro-MMP9 and shifted the collagen ratio towards collagen type III while reducing scleraxis mRNA expression by 87 % [171]. Prolonged antibiotic treatment can lead to detrimental side effects in patients, including tendinopathy, since clinically relevant doses of bactericidal antibiotics-quinolones, aminoglycosides, and β-lactams-cause mitochondrial dysfunction and ROS overproduction in mammalian cells. The deleterious effects of bactericidal antibiotics were alleviated in cell culture and in mice by the administration of the antioxidant NAC or prevented by preferential use of bacteriostatic antibiotics [215].

Melatonin as an antioxidant acts on Nrf2/HO-1 signaling pathway inhibiting ROS production and macrophage infiltration, thereby promoting tendon repair, after transplantation to the Achilles’ tendon injury site [216].

SS-31, a mitochondria-targeting antioxidant, reversed the increase in expression of MMP1, fatty acid-binding protein 4, HIF1α and proapoptotic factor Bcl2-associated protein X in primary tenocytes isolated from patients following RCT injury compared to hamstring tendon biopsy specimens [217].

### 4.2 Therapeutic approaches utilizing biomaterials with antioxidant properties

In recent years, artificial materials with antioxidant properties have been devised and increasingly applied in curative purposes in different tendinopaties. For instance, nanobiocatalysts with ferromagnetic catalytic centers that acquire a function of an artificial antioxidase with kinetics similar to catalase in ROS scavenging might be effective in protecting the stem cells pool and tissue regeneration [218]. Yet another example are cerium oxide nanoparticles with high ROS scavenging capacities. Infusion of these nanoparticles showed protective effects in a collagenase-induced tendinopathy model by activating Nrf2 [219], a transcriptional factor that provides protection against oxidative stress by balancing ROS levels [220] thus promoting tendon healing. In a rat tendon-injury model, an amalgamation of diamond-like carbon deposited on polylactic acid membranes used as an encasing of the ruptured tendon formed an anti-adhesion barrier that may reduce ROS levels effectively; also acting anitinflammatory through the NFκB inactivation and M1 polarization inhibition [211]. In support of its healing properties, this approach decelerates the release of lactic acid and its induction of macrophage M2 polarization further delaying the fibrotic process [211]. An interesting approach was made by using tannic acid (TA) to modify a decellularized tendon slice (DTS) and fabricate a functional scaffold (DTS-TA) with antioxidant and anti-inflammatory properties for tendon repair. In this setup, DTS-TA effectively reduced inflammation by increasing the M2/M1 macrophage ratio and IL4 expression, decreasing the IL6 and IL1β secretion, as well as scavenging excessive ROS in vitro and in vivo [221]. Peritendinous adhesion, secondary to the repair surgery of tendon rupture or injury, is one of the most common causes of reoperation, owing to the proliferation of fibrous tissue and excessive collagen synthesis caused by the residing inflammatory cells. Oxidative stress-responsive electrospun polyester membrane (EPM) fabricated as both physical barrier and reservoir of curcumin/celecoxib to prevent peritendinous adhesion possesses a better anti-adhesion ability compared with EPM without the drugs in addition to antioxidant properties of the multicomponent EPM [222]. An EM modification that is hailed for its anti-adhesion properties electrospun fibrous polylactic acid (PLA) membrane inhibits myofibroblast proliferation and invasion into the repaired tendon site while allowing nutrient exchange through a microporous structure [223,224]. In addition, PLA-based micro-nanofibers are also used for anti-scarring [225]. In a rat tendon-injury model, an amalgamation of diamond-like carbon (DLC) deposited on PLA membranes used as an encasing of the ruptured tendon forms an anti-adhesion barrier that may reduce ROS levels effectively; also acting anit-inflammatory through the NFκB inactivation and M1 polarization inhibition [211]. In support of its healing properties, this approach decelerates the release of lactic acid and its induction of macrophage M2 polarization further delaying the fibrosis process [211]. Although ascorbic acid is not proven as a reliable antioxidant therapeutic strategy so far, a bilayer electrospun DegraPol® fiber mesh tube infused with ascorbic acid showed a substantial amelioration of tendon injury [226]. In this approach, a pure DegraPol® layer facing the surrounding tissue minimizes fibrotic adhesions as a physical barrier, while a second layer facing the repairing tendon is infused with ascorbic acid being delivered topically to the site of the injury. Such bioactive implants may not only promote the healing process, but also mitigate oxidative stress via ROS scavenging by ascorbic acid.

A delivery of complex mixture gelatinous liposomes containing antioxidant red fluorescent carbon dots and ursolic acid exacerbated Nrf2 and HO-1 pathways showing a rescue effect in in vivo tendon injury concomitant with decreased levels of CD68, iNOS, collagen type III, αSMA, vimentin, and MMP2 [227]. Given the markers affected, this approach might be particularly efficient for the combat of adhesion after tendon injury representing a major obstacle to tendon repair, and in which oxidative stress, inflammation, and fibrosis being central to its promotion.

Sustained delivery of growth hormone-releasing hormone agonist through bovine serum albumin/heparin nanoparticles at the site of inflammation effectively reduced ROS and inhibited tendon calcification thus promoting collagen formation while preventing osteogenic differentiation in TSPCs [228].

In a rat Achilles’ tendon defect model, delivery of the enzyme mimicry nanoparticle-ceria nanozyme into the nanofiber bundle scaffold reduced oxidative damage, stabilized the mitochondrial membrane potential and ATP synthesis in TDSCs promoting structural regeneration of collagen fibers, thus recovering mechanical properties and motor function [229]. In addition, this nanozyme containing particles dysregulated immune microenvironment by alleviating senescence and apoptosis of TDSCs, downregulating the secretion of senescence-associated secretory phenotype (SASP), and inducing macrophage M2 polarization [229].

In a rat model of patellar tendon defect cerium oxide polyethylene glycol-packed nanoparticles (PEG-CeONPs) protected through their antioxidant activity human umbilical cord mesenchymal stem cells (hUCMSCs) from senescence and apoptosis under excessive oxidative stress. Transplantation of hUCMSCs loaded with PEG-CeONPs reduced ROS levels in the tendon injury area and facilitated tendon healing. Mechanistically, NFκB activator TNFα and MAPK activator dehydrocrenatine, reversed the therapeutic effect of nanoparticles indicating that they act by inhibiting the NFκB and MAPK signaling pathways [166].

As previously discussed, one of the approaches for ROS inactivation is NO delivery which might be challenging with regard to precise topical delivery to avascular dense connective tissues especially to the so-called annulus fibrosus (AF) biological barriers. NO-loaded in a modified polyvinyl alcohol and PCL-composited electrospun fiber membrane provide an excellent redox responsive milieu which is enabling programmable AF repair [230].

Use of a novel biomaterial fulleronoll in a rat collagenase tendinopathy model decreased ROS levels, inflammatory factors MMP3 and MMP13 while increasing the tendon-related factors collagen type I and tenascin C [231]. H_2_O_2_-induced ROS and inflammation in tenocytes and in vivo in a rat patellar tendon injury model were reversed by activation of the Nrf2/HO-1 pathway by delivering extracellular vesicles derived from bone marrow mesenchymal stem cells [139].

Finally, CRISPR-Cas13 technology holds substantial promise for tissue repair through its RNA editing capabilities. By employing ROS-responsive release mechanisms tailored for macrophage-targeted Cas13 RNP editing systems, the overactivation of injury-induced SPP1 (OPN encoding)-producing macrophages is curbed in the face of an acute immune microenvironment [232].

These innovative approaches mimicking natural ROS scavenging systems might be effective especially since they can be applied topically, time-sensitively and combined with different delivery systems harboring anti-tendinopathic drugs which might be their advantages over the systemic delivery of antioxidants.

## 5 Conclusions and perspectives

The survey of the literature on the role of ROS in tendon injury and repair showed us that this topic is still in its infancy. There are many studies to be conducted to arrive at the point of highly warranted meta-analyses of data that would help in defining the future directions of the field especially when it comes to therapy designation.

In many cases, the participation of ROS in tendon injury and repair was addressed circumstantially rather through the modifications of the antioxidant system and/or by an application of antioxidants than through direct manipulation of potential ROS sources such as NADPH oxidases. An additional problem is that these so-called inhibitors do not go beyond unspecific ROS scavengers that cannot pinpoint the sources from which those ROS are derived thus hampering the potential use of the selective inhibition in therapy, especially when it comes to specific NOX inhibition.

Mainly NOX1 and to a certain extent NOX4, are identified as ROS sources in tendon homeostasis and injury. However, a full panel of NOXs’ expression in the relevant tendon-specific cells is still to be conducted. ROS sources responsible for signaling in tendon stress and repair are merely explored on the level of correlation in which NOX1 and NOX4 were found upregulated in tenocytes under conditions mimicking diabetes. It might as well be that NOX1 is a primary ROS source at the early onset of tendon injury, coming both from tenocytes and endothelial cells. On the other hand, when tendon healing is in the advanced fibrotic stage, NOX4 might take over the role of the main ROS producer, as it does in many other fibrotic processes in the organism. On the other hand, the role of NOX2 and its consequent ROS signaling especially coming from myeloid sources should not be excluded.

Furthermore, upon HIF1 activation that is described in tendinopathies both NOX2 and NOX4 might be additionally upregulated since they are described as HIF-target genes [76,77].

In that respect, more studies, especially with murine tenocyte-specific deletion using Scx-Cre deleters are needed with a promising candidate being firstly NOX1, as its expression was increased in several in vitro studies in response to different stimuli. Furthermore, NOX2 deletion in myeloid-specific cells could explain the role of ROS coming from inflammatory cells for tendon tissue and repair. Besides inflammation, initial angiogenesis is the process that is important in resolving tendon injury, hence endothelial NOX deletion would help to understand the complex nature of vascular ROS’ role in these processes. However, the studies addressing the interplay between ROS derived from myeloid cells as well as vascular cells and their redox interaction with tenocytes remains completely elusive.

Furthermore, reports are suggesting that HIF1 is crucial for ECM adaptation in tendons [[72], [73], [74], [75]]. Although, as discussed in this review, there is local hypoxia building up due to depletion of oxygen following inflammation, still, probably HIF1 activation is rather a redox than a hypoxic event in tendon injury. This notion makes a compelling case for exploring ROS sources responsible for HIF1 activation in tendon injury. In line, HIF1 downstream (redox) targets might be important for further perpetuation of tendon injury and, hence, plausible therapeutic targets.

To our educated understanding, the interplay between calcium and ROS is crucial for both physiological and acute/chronic pathological responses of the tendon, but yet is grossly overlooked in the literature surveys. In support, raise of calcium levels that is documented in mechanostimulation in tendons and tenocytes speaks in favor of NADPH oxidase activation in this context since calcium is one of the upstream signaling moieties known to activate NOXs. Besides the potential activation through PKC/PKA signaling, calcium is a sole activation factor for NOX5 and DUOX1&2. However, neither NOX5 nor DUOXs were addressed in the studies so far.

A multifaceted ROS-calcium interplay forms, in our opinion, forms a self-regulating loop that keeps in check levels of both signaling species. Furthermore, the capacity of this loop for amortizing a pathological perversion either of the signaling cascades and its consequences for the mechanically overstressed tendon tissue. Calcium influx to the cell and/or its release from the intracellular depots are primary tendon responses to mechanical stress. However, increased calcium signaling, as we argue, is a key for the activation of ROS production which exert a biphasic response. Within the tendon physiological response range, following mechanical stretching, ROS propagate the signals triggered by the initial calcium increase, while on the other hand, by regulating calcium levels, ROS keep those very signals in check. In this way, a physiological role of ROS in the tendon physiology could be envisaged also as a calcium signaling limiting. However, as we understand, in frequently encountered pathological conditions in the tendon, inter-regulation between calcium and ROS is lost and it rather results in a harmful positive feedback loop which impairs tendon recovery.

Finally, it would be beneficial to understand if there is a measurable rescue when it comes to tendon injury especially in combination with specific NOX subunit impairment. One of the central novel concepts explored in this review ROS-calcium axis central novel concept that this review offers, opening the avenues for a deeper understanding of the calcium-ROS interplay in the mechanical tendon injury, thus identifying new therapeutic targets in either sole or combinational therapies.

This could potentially open a new line of research linking mechanical overload to ROS-induced tendinopathy, possibly through calcium and NADPH oxidases.

## CRediT authorship contribution statement

**Damir Kračun:** Writing – review & editing, Writing – original draft, Conceptualization. **Agnes Görlach:** Writing – review & editing. **Jess G. Snedeker:** Writing – review & editing. **Johanna Buschmann:** Writing – review & editing, Resources, Funding acquisition, Conceptualization.

## Funding

This study was supported by Swiss National Scientific Foundation (SNSF) (“Tendon Healing” 310030_197578) to JB and (“Tension regulation and dysregulation in tendon healing and fibrosis“ 207489) to JS.

## Declaration of competing interest

The authors declare that they do not have any financial or other competing interests.

## Data Availability

No data was used for the research described in the article.
